# Spatial ecology of male hippopotamus in a changing watershed

**DOI:** 10.1038/s41598-019-51845-y

**Published:** 2019-10-28

**Authors:** Keenan Stears, Tristan A. Nuñez, Epaphras A. Muse, Benezeth M. Mutayoba, Douglas J. McCauley

**Affiliations:** 1 Department of Ecology, Evolution and Marine Biology & Marine Science Institute, University of California, Santa Barbara, California, 93106 USA; 2 Department of Zoology and Physiology, University of Wyoming, Laramie, Wyoming 82071 USA; 3 Tanzania National Parks Authority, Ruaha National Park, P.O. Box 369, Iringa, Tanzania; 4 Department of Veterinary Physiology, Biochemistry and Pharmacology, Sokoine University of Agriculture, P.O. Box 3017, Morogoro, Tanzania

**Keywords:** Animal migration, Behavioural ecology, Climate-change ecology, Conservation biology, Animal behaviour

## Abstract

The obligate dependency of the common hippopotamus, *Hippopotamus amphibius*, on water makes them particularly vulnerable to hydrological disturbances. Despite the threats facing this at-risk species, there is a lack of information regarding *H. amphibius* spatial ecology. We used high-resolution tracking data of male *H. amphibius* to assess home range size, movement mode (e.g. residency and migratory movements), and resource selection patterns. We compared these results across seasons to understand how hydrological variability influences *H. amphibius* movement. Our study watershed has been severely impacted by anthropogenic water abstraction causing the river to stop flowing for prolonged periods. We observed *H. amphibius* movements to be highly constrained to the river course with grassy floodplains being their preferred habitat. Dominant and small sub-adult males displayed year-round residency in/near river pools and had smaller home ranges compared to large sub-adults. During the dry season, large sub-adult males made significant (~15 km) upstream movements. The larger home range size of large sub-adults can be attributed to the elevated levels of migratory and exploratory activities to limit conspecific aggression as the river dries. Our observations provide insight into how future changes in water flow may influence male *H. amphibius* movements and populations through density-dependent effects.

## Introduction

In African savannas, the common hippopotamus (*Hippopotamus amphibius*) is an important ecosystem engineer because it shapes the physical structure of ecosystems^1^, vegetation communities^2,3^, and biogeochemical cycling^4,5^. These effects are detectable in both aquatic and terrestrial ecosystems. Across their range, *H. amphibius* populations are declining due to a variety of factors including habitat loss and degradation as well as illegal and unregulated hunting practices^6^.

The semi-aquatic nature of *H. amphibius* makes it highly vulnerable to human-driven hydrological change^7,8^. Suitable water availability is essential for *H. amphibius* for two reasons. Firstly, the unique skin of *H. amphibius* is susceptible to cracking when exposed to direct sunlight; for the skin to maintain its thermoregulatory function, it needs regular immersion in water^9^. Secondly, Clauss, *et al*.^10^ posit that the reliance of *H. amphibius* on water is a consequence of high faecal water loss related to their gastrointestinal morphology. Intensified anthropogenic water abstraction and the associated drying of lakes and rivers, in addition to accelerating rates of agricultural and urban development along riverine and lacustrine environments, are major sources of direct and indirect stress for *H. amphibius*. Furthermore, climate change-associated shifts in rainfall, which affect watershed hydrology through prolonged drought and intensified rainfall events, appear to be creating conditions that amplify this stress^5^.

Seasonal drying events that reduce river flow can naturally limit the number of aquatic refugia (e.g. deep river pools) for *H. amphibius* and can cause large, densely packed aggregations of *H. amphibius* to form within remaining pools^5,11^. This dry season crowding can become exacerbated under conditions where river discharge has been significantly reduced due to anthropogenic water abstraction. Stress within *H. amphibius* populations during these prolonged dry periods can be further elevated as a result of increased competition. For example, dominant male *H. amphibius* are territorial and defend aquatic refugia and can be extremely aggressive to sub-adult male *H. amphibius*, frequently ejecting them from pools, thereby forcing these displaced individuals to disperse to find their own pool, often of lower quality^7^. In addition, during their nightly foraging bouts, *H. amphibius* consume approximately 40–50 kg wet mass^12^, thus requiring  productive terrestrial habitats that can support their foraging needs. These resources become more difficult to obtain during dry seasons – and more so in dry seasons where vegetation is stressed by human activity (e.g. livestock grazing). Thus, it is critically important to understand how the spatial and temporal structuring of vital aquatic and terrestrial resources influence the spatial ecology of *H. amphibius*. The mechanisms that drive *H. amphibius* movements can influence their fitness as well as regulate the aforementioned effects that *H. amphibius* have upon communities and ecosystems e.g.^13,14^.

To date, very little is known about the spatial ecology of *H. amphibius*. There is a paucity of information available on the core patterns of *H. amphibius* spatial ecology and the drivers that shape these patterns of space use. Generating information of this kind is essential for improving our understanding of the ecology of *H. amphibius* and can also be used to develop more spatially informed and, thus, more effective conservation and management strategies for this at-risk species. Such information is needed more than ever in this period of rapid environmental change^15,16^.

To address these issues, we used GPS technology to track male *H. amphibius* movements in a historically perennial river in central Tanzania that, as a result of human modification, now dries down seasonally into a series of isolated pools. GPS devices were deployed for one year, covering multiple seasons, which allowed us to elucidate how seasonal variability in both terrestrial resources and aquatic refugia influenced *H. amphibius* movements. From these data, we asked two broad questions: (1) what are the spatial patterns by which male *H. amphibius* use their landscape? and (2) how does hydrological variation shape these patterns of male *H. amphibius* space use? We focused our tracking efforts on male *H. amphibius* because their movements are more likely to be influenced by water availability compared to female *H. amphibius*. These differences are potentially due to the interaction between altered water availability and the social structure of this species (i.e. increased competition and aggression between males as suitable habitat declines)^11^. Collectively, the results from our study contribute the  first high-resolution view of the spatial ecology of male *H. amphibius* and elucidate the high degree of sensitivity of their movements to environmental change.

## Methods

### Study area

**Figure 1:**
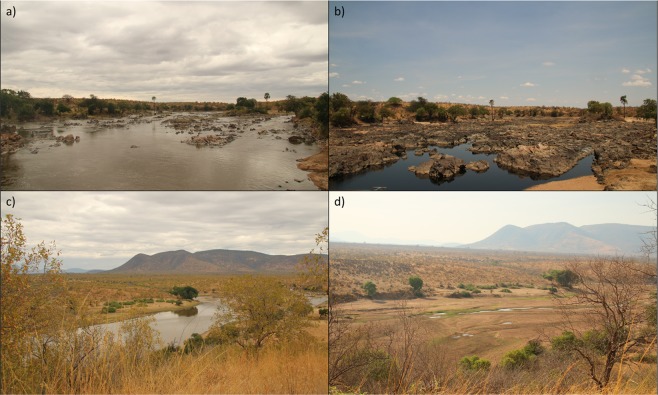
Comparison of wet and dry season river flow in the Great Ruaha River from two vantage points. Panels (**a,c**) depict when the river is flowing during the wet season and (**b,d**) shows when river flow ceases and only isolated pools remain during the dry season.

This study was conducted in the Ruaha National Park in central Tanzania (7°42′ S, 34 °54′ E) from November 2016 to  December 2017. Ruaha National Park encompasses a transitional vegetation zone between the East African *Acacia-Commiphora* zone and the Southern African *Brachystegia* and *Miombo* zone^17,18^. Mean annual rainfall in this region is approximately 580 mm with most rainfall occurring during the wet season from November/December to May. The extensive dry season spans from June to November/December^19^. From 1960–1990 the Great Ruaha River flowed throughout the year and maintained 1–3 m^3^ s^−1^ dry season flow^20^. However, from 1993 to present day (2019), intensive water abstraction from the Great Ruaha River by agriculture has consistently reduced dry season river flow to zero^20^. As a result, approximately 60% of the river dries up^19^ and only discrete pools remain that are separated by large expanses of dry river bed (Fig. 1).

### Hydrological monitoring

Monthly rainfall records for Ruaha National Park during our study period were obtained from park officials. River hydrology was monitored at the Msembe gauging station located ~2 km upstream of our sampling area. For the purpose of our study, we partitioned our *H. amphibius* movement data into five seasonal periods defined as follows based on rainfall and river hydrology: (1) Peak dry period: no rainfall and zero flow, (2) Wetting period: start of the rainy season with an increase in river flow only being observed towards the end of the wetting period, (3) Peak wet: peak rainfall and river flow, (4) Drying period: no rainfall and rapidly declining river flow, and (5) Second peak dry: no rainfall and zero flow (Supplementary Information Fig. S1). Our study period consists of two dry periods; however, our goal was to quantify seasonal, rather than inter-annual variation in *H. amphibius* movement. Consequently, for the below analyses, we combined observations from these two periods into a single dry season.

### *H. amphibius* GPS tracking

All *H. amphibius* were immobilized in strict accordance with the guidelines of the American Society of Mammalogists for the use of wild mammals in research. The protocol was approved by the Institutional Animal Care and Use Committee at the University of California Santa Barbara (protocol number 914). Tanzania National Parks Authority veterinarians conducted the immobilizations and approval was received by the Tanzanian Wildlife Research Institute and the Tanzania Commission of Science and Technology (permit numbers 2016–286-ER-2013-52 and 2017–331-NA-2013-52).

We tracked 10 male *H. amphibius* in the Great Ruaha River using GPS-GSM UHF collars (Wireless Wildlife, Potchefstroom, South Africa). The dry conditions of our study site allowed veterinarians to immobilize *H. amphibius* away from water sources using a gas-propelled dart, following protocols outlined in^21,22^. Due to the difficulties in attaching a collar to the neck of *H. amphibius*^7^, we fitted a modified rhinoceros ankle collar to the front foot of *H. amphibius*. After the reversal drug was administered, we observed each *H. amphibius* for ~1 hr to ensure that (1) the collar did not influence their normal behaviour, and (2) each *H. amphibius* was able to safely return to its river pool. No complications were observed for any of the collared individuals.

Collars were programmed to acquire a location fix every 30 minutes between 18h00 and 06h00. The start and end of the GPS sampling period was based on observing camera trap images noting the time that *H. amphibius* left the river to forage and the approximate time many returned to the river after each nightly foraging bout. As a result, the assessment of *H. amphibius* spatial ecology is based on the nocturnal movement patterns while foraging. We used natural breaks in the distribution of body length (tip of snout to base of the tail) measurements of tracked *H. amphibius* to define three life stage categories: “dominant male”, “large sub-adult male”, and “small sub-adult male” (Supplementary Information Fig. S2).

Given the fix-rate, collars were expected to last approximately one year. However, during the study period, some collars did fall off the ankle of *H. amphibius* before the end of the one year period. Seasonal sample sizes for each life stage category used in the below analyses are provided in Table 1 (See Supplementary Information Table S1 for a timeline showing the duration that each collar collected location data as well as the total number of fixes). These collar losses were not associated with *H. amphibius* injury or death, but rather because of the difficult conditions that the collars were subjected to (e.g. submerged under water for extended periods). Finally, we conducted stationary experiments to assess GPS collar fix-success rate and location error (see Supplementary Method S1)^23^. The fix success rate was 0.99 with only 8 missed fixes out of the 953 fix attempts during the test period and the location error for the collars ranged from 8–17 m (average: 11 m).

**Table 1:** The number of *Hippopotamus amphibius* that were tracked in each life stage category per season.

Life stage	Season				
	Peak dry	Wetting	Peak wet	Drying	Second peak dry
Dominant male	4	4	3	3	3
Large sub-adult male	2	2	2	2	2
Small sub-adult	4	4	2	2	2
Total	10	10	7	7	7

### *H. amphibius* space use

#### *H. amphibius* home range

We estimated *H. amphibius* home ranges using the Time Local Convex Hull approach implemented in the R package, ‘T-LoCoH’^24^, which takes into account both spatial and autocorrelation of GPS fixes when creating convex hulls. For hull construction, T-LoCoH uses a distance function that transforms a unit of time into a unit of distance, called the time-scaled distance TSD^25^. We weighted the time and space components of the TSD by setting the scaling parameter (*s*) to a time interval of interest. We selected a time interval of five hours based on our observations of the average duration of a nightly foraging bout for *H. amphibius*, which is similar to the estimated 30% of the day they spend feeding^26^. To make comparisons across individuals and seasons, we used the same approach for all individuals in each season by always estimating the scaling parameter at five hours. Finally, we used the *k*-method of sampling to construct polygons^25^. To ensure the selected value of *k* did not result in a sudden increase in area used by an individual *H. amphibius*, we assessed the isopleth area curves and compared the perimeter: area estimates.

We explored the factors that shape *H. amphibius* home range estimates using a generalized linear mixed effect model (gamma error distribution and log-link function) using the ‘lme4’ package in R^27^. For these models, we included season, life stage, and their interaction as main effects. We used individual *H. amphibius* as a random grouping effect. We found a significant interaction effect; therefore, we conducted a Tukey’s pairwise post-hoc analyses of marginal means to elucidate differences between the interaction terms.

#### Relationship between distance from the river and key foraging areas

Given that the Great Ruaha River appeared to play a key role in shaping *H. amphibius* movement in this semi-arid environment, we analysed several attributes that aided in determining the relationship between *H. amphibius* and the river. Specifically, we used T-LoCoH’s revisitation rate and duration of visitation to delineate important foraging areas within individual home ranges. Revisitation rate (nsv) is the number of separate visits to the area inside an individual polygon and the average duration of each visit (mnlv) is the number of GPS locations per visit to an individual polygon. We calculated the revisitation rate and duration of visits for each hull based on an inter-visit gap (IVG) of five hours (the average duration of a nightly foraging bout). Thus, separate visits to a hull were identified when an individual *H. amphibius* left a given hull and only returned after a period of 5 hours.

To relate revisitation rate and visit duration (proxy for important foraging areas) with distance travelled away from the river, we calculated and extracted the distance between the Great Ruaha River and every *H. amphibius* GPS location using the ‘gdistance’ function in the R package, ‘rgeos’^28^. We used Kendall’s rank correlation coefficient to determine if distance from the river was correlated with high or low occurrences of revisitation and visitation duration. We explored this relationship for each season (See Table 1 for sample sizes). Furthermore, we ran generalized linear mixed models (gamma error distribution and log-link function) to determine whether the mean distance travelled away from the river by *H. amphibius* was influenced by season, life stage, or their interaction. We included individual *H. amphibius* as a random grouping effect. We found a significant interaction effect; therefore, we conducted a Tukey’s pairwise post-hoc analyses of marginal means.

#### Characterization of *H. amphibius* movement modes

To classify *H. amphibius* movement modes, we integrated distributions of net squared displacement values (NSD) calculated from GPS fixes from the duration of the study with latent, discrete-state models using the ‘lsmnsd’ package in R^29^. NSD is the square of the Euclidean distance between a starting location and each subsequent location. The latest, discrete-state models (a type of hidden Markov model) allow for greater flexibility and accuracy in classifying large-scale movement modes^30,31^. These models define movement modes based on the distribution of NSD. For example, normally distributed NSD values are associated with areas of intensive and recurrent space use (e.g. resident) whereas a uniform distribution is indicative of sporadic use of an area while travelling (e.g. dispersing). We defined the starting location as the initial pool in which the respective *H. amphibius* was collared. For each individual (n = 10), we characterized movement modes by extracting the switching probability and the number of transitions between movement modes identified by the model (for further details see^29^). For brevity, we only present a single figure for each of the different movement modes that we observed (see Supplementary Information Fig. S3 for the NSD plots and switching probabilities for the different movement modes for the remaining individuals). We overlaid these NSD values with season to identify potential mechanisms driving individual movement modes. Furthermore, we calculated modified NSD metrics that measured displacement along the river and away from the river (See Supplementary Method S2).

### *H. amphibius* habitat selection

#### Environmental data

We obtained spatial data on vegetation cover types and the distance to the Great Ruaha River (m) from the Ruaha National Park GIS landcover type database. The landcover and vegetation map was derived from Landsat Thematic Mapper satellite imagery using unsupervised classification algorithms^32^. Vegetation types were mapped to a 30 m resolution and the reliability of the vegetation cover was assessed by comparing map classification with field measurements. From the landcover map, we defined and included the following vegetation cover types in our models: floodplain (area within the banks of the Great Ruaha River), drainage lines (small seasonal streams that flow into the Great Ruaha River), short grass savanna (open savanna dominated by short grass with ~15% shrub cover), tall grass savanna (open savanna dominated by tall grass with ~15% shrub cover), shrub dominated savanna (40–65% shrub cover), and tall tree savanna (40–65% tree cover with the understory dominated by short grass). We derived slope estimates (degrees) from high-resolution satellite imagery (Planet satellite imagery) of the study site by creating a digital elevation model (DEM) and extracting slope values in QGIS^33^. All datasets were converted to 30 m resolution using the ‘raster’ package in R^34^. We assessed for collinearity among environmental data variables using Pearson correlation coefficients. We found that distance from the Great Ruaha River, distance from anthropogenic settlements (e.g. camps), and distance from roads were highly correlated (r > 0.6). From these variables, we only included distance from the Great Ruaha River in our resource selection functions described below because it was a better predictor of *H. amphibius* locations compared to distance from anthropogenic settlements and distance from roads.

#### Resource selection functions

We fitted seasonal RSFs using generalized linear mixed effects models with a binomial error distribution and log-link function. We included random intercepts and random slope coefficients to account for unequal sample sizes and individual-specific differences in habitat selection^35^. Within the home range of each individual *H. amphibius*, we paired used locations with randomly generated available locations selected from within the 100% minimum convex polygon (i.e. third-order selection^36^). To reduce bias and improve the interpretation of coefficients obtained from RSF models, a sufficiently large sample of available points needs to be generated and the spatial extent of these available points must match the scale of inference over which habitat selection is being inferred (in this case, third-order selection)^37,38^. Thus, following Fithian and Hastie^39^, we weighted the availability of randomly selected available points so that there were 5 times more available locations than used locations. Furthermore, the available points were generated from the same spatial extent as the used locations (i.e. both used and available locations were obtained from within the 100% minimum convex polygon).

Prior to model selection, RSFs were partitioned into two life stage categories. For the first category, we combined dominant males with small sub-adult males because of their similar home ranges and movement modes (i.e. dominant males tolerate sub-adult males permitting overlap between their spatial ecology). For the second category, we analysed large sub-adult males separately because of the stark contrast in home range and movement modes compared to dominant and small sub-adult males. For each season (peak dry, wetting, peak wet, and drying), we ran seven different models where we regressed *H. amphibius* habitat use by the abovementioned environmental data (Supplementary Information Tables S2 and S3). We selected the best-performing model using AICc scores and Akaike weights^40^. For categorical environmental variables (e.g. vegetation cover), preference was modelled in respect to a reference category^37^. We selected floodplain habitat as the reference category because it was the habitat that was consistently preferred (i.e. positive selection ratios; Fig. S5). Finally, we determined the ability of each life stage-specific seasonal RSF model to predict *H. amphibius* habitat selection using k-fold cross validation^41^.

## Results

### *H. amphibius* home range

**Figure 2:**
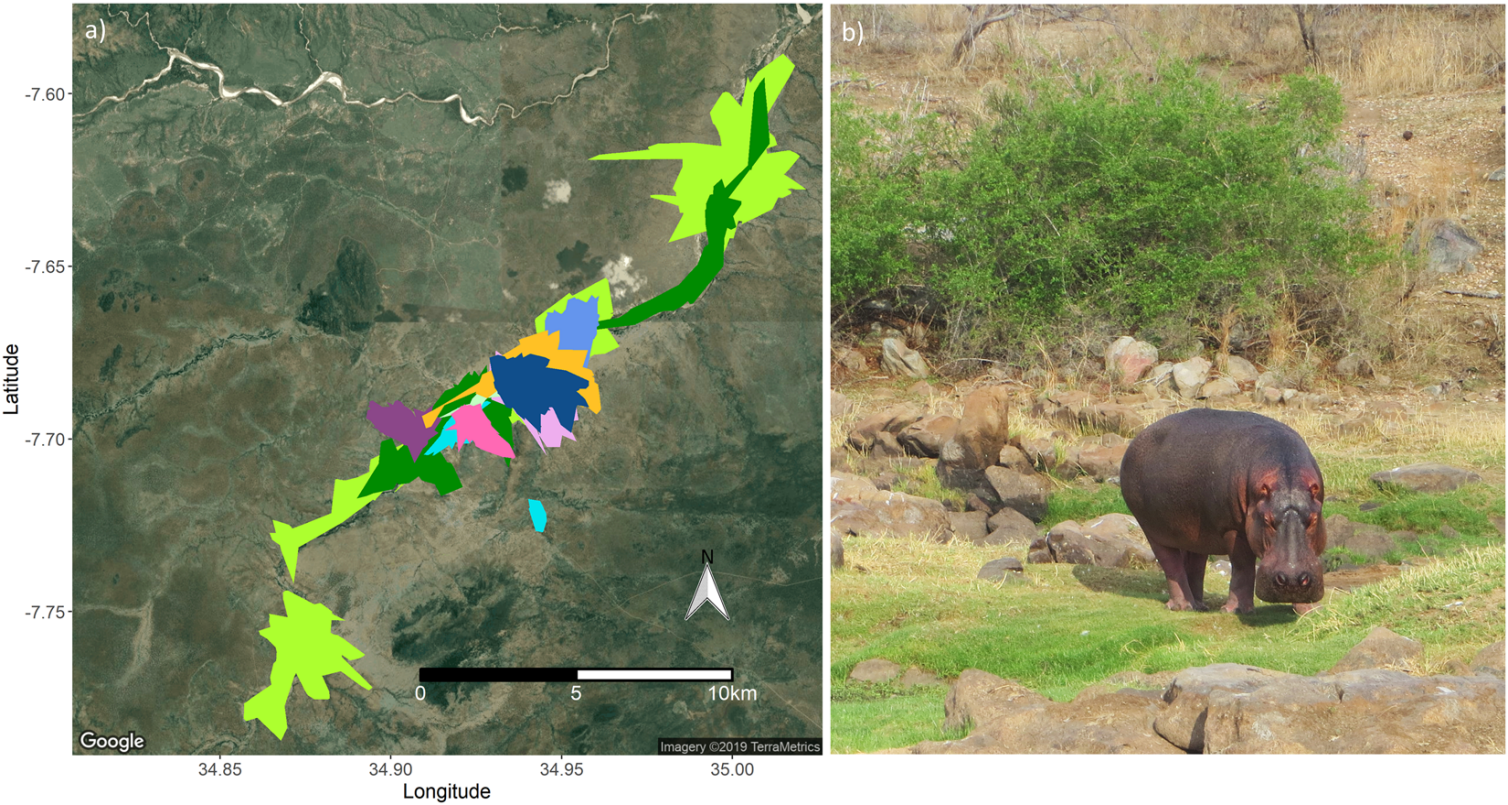
(**a**) Long-term home ranges (95% utilization distribution) for all male *Hippopotamus amphibius* (*n* = 10) along the Great Ruaha River, Ruaha National Park, Tanzania (November 2016 to December 2017). Each individual is represented by a different colour. (**b**) *H. amphibius* grazing on green grass within its preferred habitat (floodplains) during the dry season.

**Figure 3:**
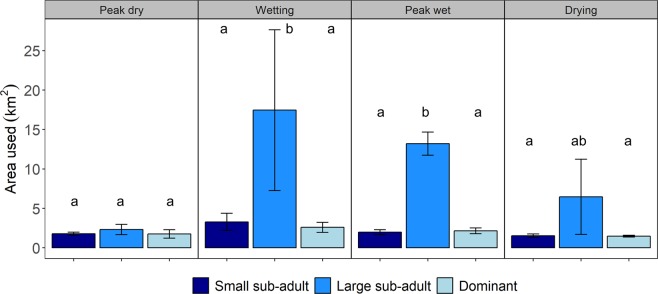
Home range size (mean ± SE) of male *Hippopotamus amphibius* calculated using the 95% density isopleth (utilization distribution). Data is separated by three different *H. amphibius* life stages and analysed within the context of four different seasons (peak dry and wetting: *n* = 4 dominant males, *n* = 2 large sub-adult males, and *n* = 4 small sub-adult males; peak wet and drying: *n* = 3 dominant males, *n* = 2 large sub-adult, and *n* = 2 small sub-adults). Letters denote significant differences (*P* < 0.05) in home range size used by the different *H. amphibius* life stages.

When pooling all data from this study, we estimated that *H. amphibius* in this system occupied a home range averaging 8 ± 3 km^2^ (±SE) (individual range: 1.6–37.6 km^2^; Fig. 2). These home range sizes varied by season and *H. amphibius* life stage (χ^2^ = 17.7, df = 6, *P* = 0.007; Fig. 3). Large sub-adult males traversed home ranges that were more than three times larger than other individuals during the wetter parts of the year (Fig. 3). After an initial increase in home range size following the first dry season, dominant males maintained relatively constant home ranges throughout the year. Changes in water availability did not affect the home range size of dominant and small sub-adult males. Comparatively, these two life stages maintained similar home ranges throughout the study (*P* > 0.05; Fig. 3).

### Relationship between distance from the river and key foraging areas

**Figure 4:**
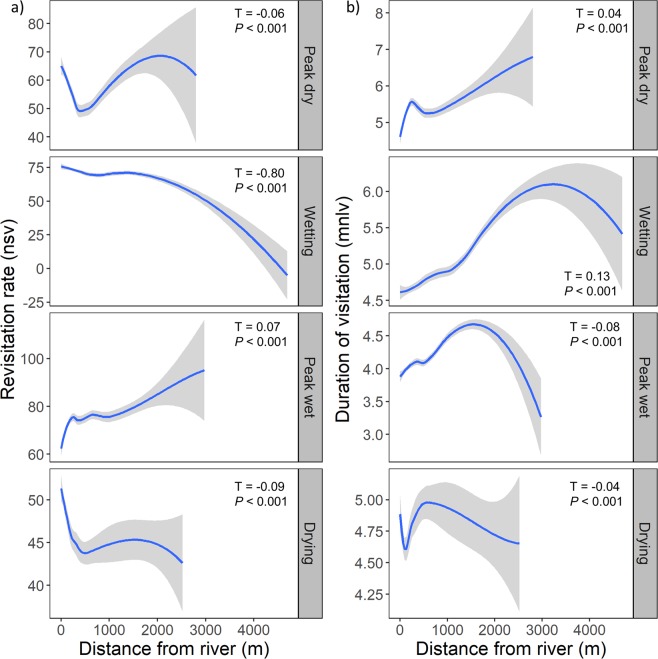
Kendall’s rank correlation coefficient (Τ) showing the relationship between (**a**) revisitation rate (mean number of separate visits to a given hull, nsv), and (**b**) visit duration (mean number of locations per visit, mnsv) and the distance *Hippopotamus amphibius* (*n* = 10) travelled from the Great Ruaha River in each of the four seasons. Best-fit lines were generated using the LOESS smoothing method.

We defined key foraging areas for *H. amphibius* as hulls that experienced high revisitation rates. These key foraging areas were highly structured around the river with revisitation rates decreasing as the distance from the river increased. We observed this trend in all seasonal periods, except for the peak wet season where we found an inverse trend (Fig. 4a). In addition, *H. amphibius* spent more time in areas further away from the river. We observed this pattern in all seasons, except the peak wet and drying periods (Fig. 4b).

**Figure 5:**
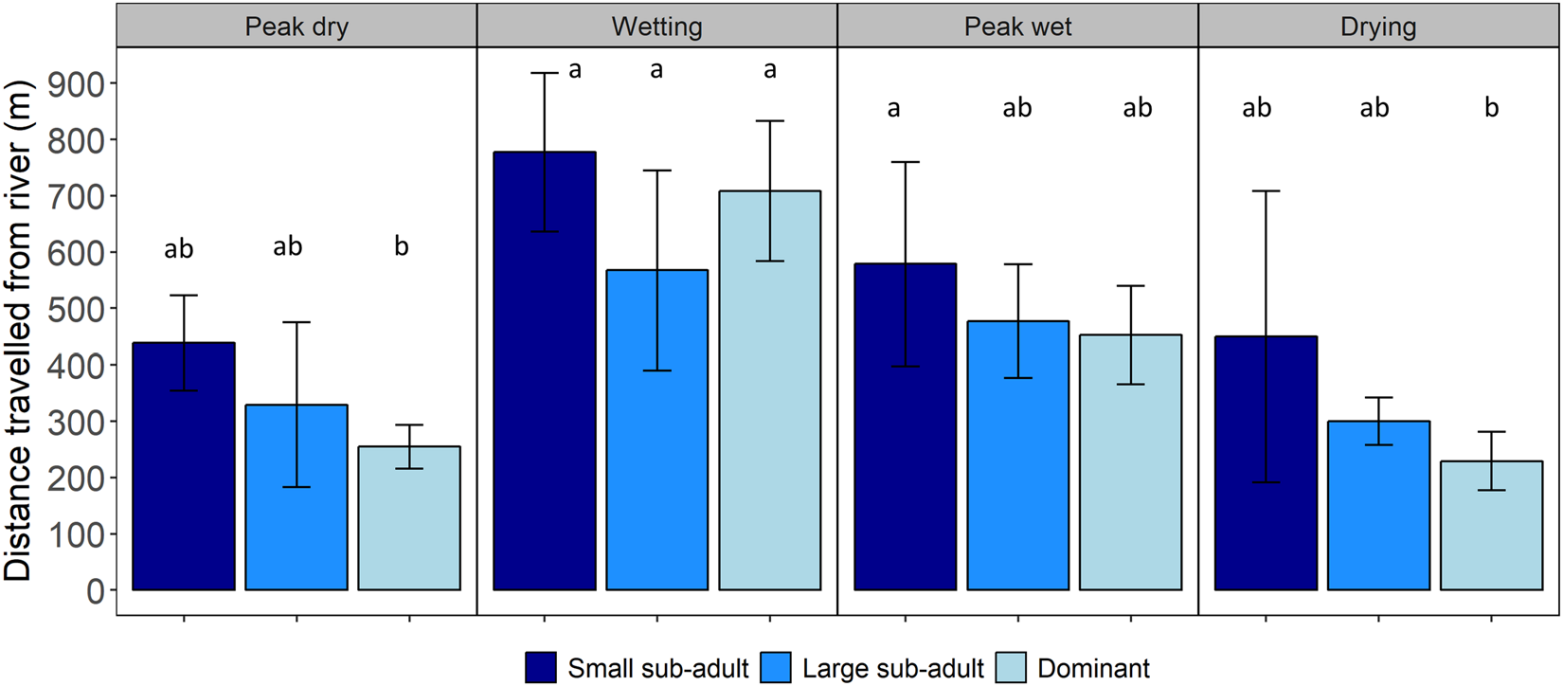
Distance travelled away from the Great Ruaha River (mean ± SE) by the different life stage categories of male *Hippopotamus amphibius* in each of the seasonal sampling periods (peak dry and wetting: *n* = 4 dominant males, *n* = 2 large sub-adult males, and *n* = 4 small sub-adult males; peak wet and drying: *n* = 3 dominant males, *n* = 2 large sub-adult, and *n* = 2 small sub-adults). Letters denote significant differences (*P* < 0.05) in mean distance travelled away from the river obtained by the different *H. amphibius* life stages.

Throughout the entire sampling period, the average distance and the average maximum distance travelled away from the river by *H. amphibius* was 0.5 ± 0.003 km (individual average range: 0.2–0.7 km) and 1.9 ± 0.1 km (individual average range: 1.3–2.5 km), respectively. The absolute maximum distance any individual *H. amphibius* travelled from the river was 4.7 km. We found a significant interaction between season and *H. amphibius* life stage in respect to the distance travelled away from the river (χ^2^ = 12.632, df = 6, *P* = 0.05; Fig. 5), which was primarily driven by an increase in the distance travelled away from the river by dominant males during the wetting and peak wet periods. Post-hoc comparisons revealed that within each season, there were no differences in the distance travelled away from the river among the three *H. amphibius* life stage categories (*P* > 0.05).

### Characterization of *H. amphibius* movement modes

The variation in *H. amphibius* home range size appears to be attributable to the different individual movement modes that we modelled. We classified two distinct movement modes for large sub-adult males. Both these movement modes involved large-scale movements within or parallel to the river, rather than movements perpendicular to the river (Fig. S4).

**Figure 6:**
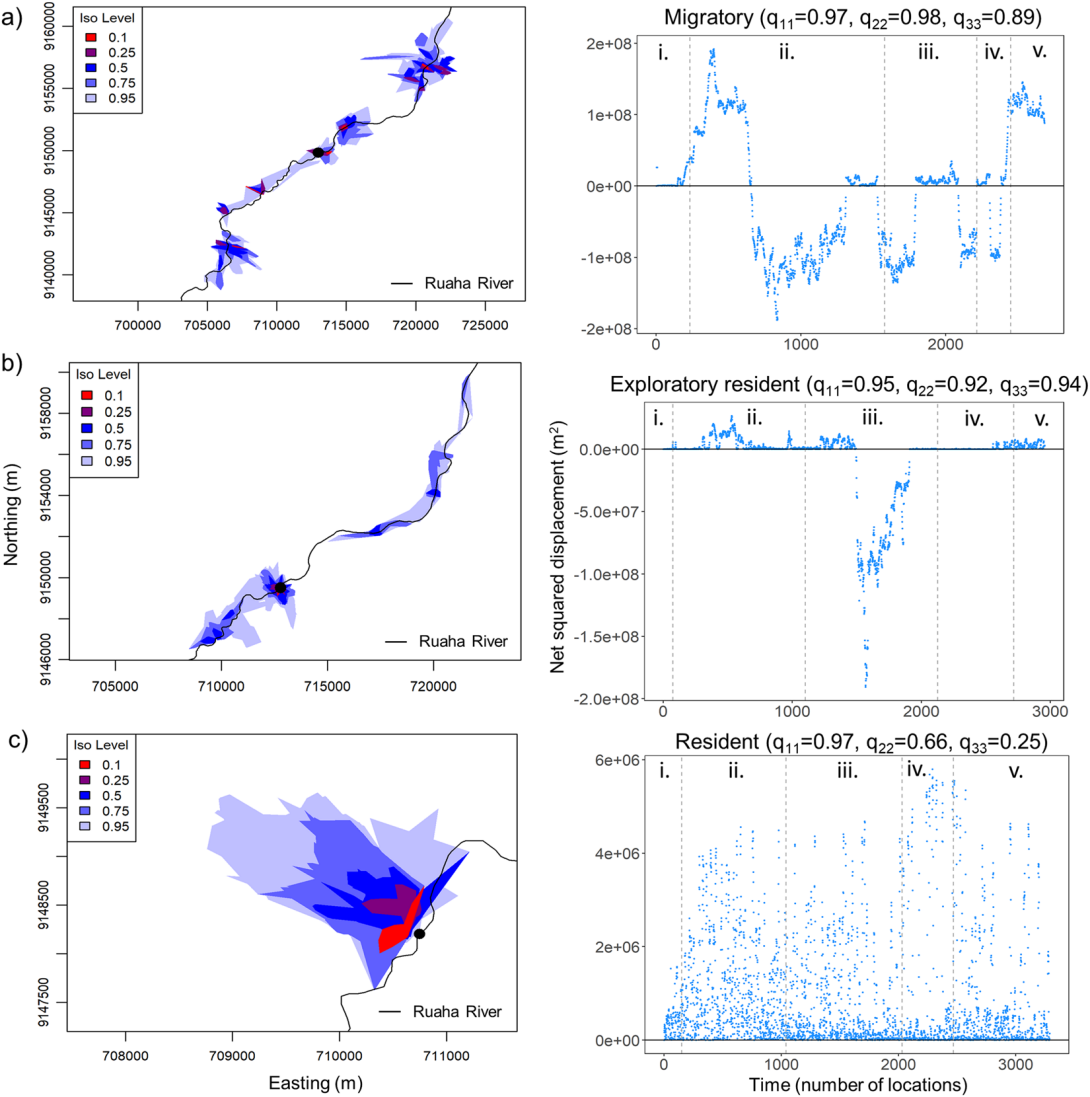
Examples of *Hippopotamus amphibius* movement data and the corresponding pattern in net squared displacement for *H. amphibius* that match (**a**) migratory movement patterns, (**b**) residency and exploratory movements, and (**c**) residency around an aquatic refuge. Within *H. amphibius* home ranges, red denotes areas of high use and the black point represent the initial pool in which the individual was collared. Iso level refers to the density of isopleths (i.e. 0.95 reflects the 95% home range). Switching probabilities (q_11_, q_22_, and q_33_) that were used to classify movement modes are also presented. Positive net squared displacement values reflect upstream movement while negative values denote downstream movement of *H. amphibius* from their starting pool. Net squared displacement values are plotted across seasons in the second column (dashed grey line): (i) peak dry, (ii) wetting, (iii) peak wet, (iv) drying, and (v) second peak dry.

One large sub-adult male showed patterns of transition between two movement modes indicative of migratory behaviour (switching probabilities for migration when: q_11_ > 0.90, q_22_ > 0.90, and q_33_ > 0.85; Fig. 6a). This individual moved between multiple core areas within the sampling period. Upstream migrations (range: 3–15 km from the initial pool) occurred during the driest parts of the year and lasted ~60 days, whereas downstream migrations (range: 2–15 km below the initial pool) occurred during wetter periods of the year and lasted ~81 days (Fig. 6a).

The second large sub-adult male also showed a pattern of transition between movement modes similar to a migratory pattern. However, this individual spent less time in the second movement mode relative to the first movement mode. As such, a movement pattern akin to exploratory resident is the most appropriate movement mode classification (Fig. 6b). This individual remained resident around its initial pool, but explored two core areas throughout the sampling period. This individual showed similar patterns when compared to the other large sub-adult by moving upstream (range: 2–5 km and lasting 60 days) during dry periods and moving downstream (range: 5–15 km lasting 38 days) during wet periods of the year.

In contrast, dominant and small sub-adult males did not exhibit large-scale up or downstream movements (Supplementary Information, Fig. S4). In fact, they showed highly constrained movements around their respective pools that is indicative of residency (switching probabilities for resident movement strategy when: q_22_ ≤ 0.90 and q_33_ ≤ 0.90; Fig. 6c).

### *H. amphibius* habitat selection

All top seasonal RSF models had relatively high accuracy in predicting *H. amphibius* occurrence: dominant and small sub-adult *H. amphibius* accuracy range: 61–80%; large sub-adult accuracy range: 67–77%. Seasonal RSF models revealed that all male *H. amphibius* selected for areas closer to the river (distance from river coefficients), except during the wetting period where dominant and small sub-adult males selected for areas further from the river (Table 2). Dominant and small sub-adult males selected for less steep areas in all seasons except the peak dry period when they selected for steeper areas. Large sub-adults preferred less steep areas during the wetting period, but preferred steeper areas during the peak wet and drying periods.

**Table 2:** Seasonal coefficient estimates from the top performing resource selection models for *Hippopotamus amphibius*.

Season	Dominant and small sub-adult males				Large sub-adult males		
	Coefficient	Beta	SE	P	Beta	SE	P
Peak dry	Intercept	−9.03	0.53	<0.001	−9.23	0.18	<0.001
	Distance from river	−0.99	0.27	<0.001	−0.38	0.19	0.043
	Drainage	−0.46	0.64	0.4986	−0.39	1.34	0.77
	Short grass	−0.56	0.24	0.1268	−0.3	0.38	0.434
	Shrub	−1.8	0.34	0.0007	−0.77	0.47	0.095
	Tall grass	−0.59	0.35	0.1223	−0.71	0.33	0.03
	Tall tree	−1.32	1.06	0.6523	−0.51	1.78	0.772
	Slope	0.01	0.12	0.1446	—	—	—
Wetting	Intercept	−7.98	0.31	<0.001	−8.21	0.34	<0.001
	Distance from river	0.16	0.32	0.621	−0.65	0.14	<0.001
	Drainage	−0.32	0.33	0.327	0.02	0.56	0.972
	Short grass	−0.73	0.24	0.002	0.16	0.34	0.639
	Shrub	−1.51	0.3	<0.001	−0.78	0.16	<0.001
	Tall grass	−1.14	0.23	<0.001	0.07	0.22	0.748
	Tall tree	1.28	1.66	0.441	−0.48	0.57	0.402
	Slope	−0.23	0.07	0.003	−0.06	0.13	0.665
Peak wet	Intercept	−8.23	0.19	<0.001	−8.95	0.31	<0.001
	Distance from river	−0.78	0.24	<0.001	−1.01	0.07	<0.001
	Drainage	0.23	0.24	0.321	0.68	0.48	0.160
	Short grass	−0.3	0.23	0.179	0.05	0.11	0.673
	Shrub	−0.47	0.27	0.074	0.07	0.36	0.852
	Tall grass	−1.14	0.25	<0.001	0.14	0.48	0.772
	Tall tree	10.55	3.28	<0.001	0.65	0.12	<0.001
	Slope	−0.09	0.13	0.524	—	—	—
Drying	Intercept	−8.62	0.41	<0.001	−9.28	0.18	<0.001
	Distance from river	−1.41	0.46	0.002	−0.85	0.24	<0.001
	Drainage	−1.58	0.46	<0.001	−1.65	1.68	0.327
	Short grass	−1.10	0.28	<0.001	−0.57	0.13	<0.001
	Shrub	−2.54	0.33	<0.001	−2.29	0.32	<0.001
	Tall grass	−2.81	0.43	<0.001	−1.00	0.66	0.132
	Tall tree	−0.67	0.32	0.037	−0.92	0.70	0.188
	Slope	−0.20	0.06	0.331	0.18	0.06	0.001

The floodplain habitat was used as the reference category for all models and the use of other habitats is relative to the reference category.

Patterns of *H. amphibius* selection for different habitats appeared to be strongly influenced by season. During the peak dry and drying periods, *H. amphibius* used all habitats less than expected when compared to floodplains (Table 2). However, when rainfall and river flow increased (wetting and peak wet periods), all *H. amphibius* moved out of the floodplains and selected for specific upland habitats. Dominant and small sub-adult males selected for tall tree habitats (both wetting and peak wet periods) and drainage lines (peak wet period). Large sub-adults, selected for drainage lines (wetting and peak wet periods), short grass habitats (wetting and peak wet periods), tall grass (wetting period only), and tall tree habitats (peak wet).

## Discussion

Our findings represent the first high-resolution data on *H. amphibius* movement and insight into the mechanisms that shape their spatial ecology. Previous research on *H. amphibius* spatial ecology has been based upon directly observing *H. amphibius* as they move across landscapes and manually following *H. amphibius* foraging paths^42,43^. In addition, our  results provide insight into how variation in river flow may influence the core patterns of *H. amphibius* movement and habitat use and which *H. amphibius* are most affected by this variation.

Home range size and the distance travelled away from the river by *H. amphibius* are particularly useful core measures to better understand the basic principles for protected area design strategy for the numerous sub-Saharan African parks that host populations of *H. amphibius.* Futhermore, these measures can be used  to gauge the potential for *H. amphibius*-human conflict in *H. amphibius*-inhabited riverine areas. On average, the resident and migratory *H. amphibius* we tracked occupied a home range of ~3 km^2^ and 26 km^2^ (averaging ~8 km^2^), respectively during the course of our study, which is considerably smaller than other African megaherbivores such as white rhinoceros, *Ceratotherium simum* ~0.75–45 km^2^ ^44,45^ and elephant, *Loxodonta Africana* ~200–10,000 km^2^ ^46,47^. In comparison, the smaller home ranges of *H. amphibius* likely derives from the need of this obligate aquatic mammal to return to their aquatic refuges daily.

Tracking data also helped to quantify the distances that *H. amphibius* move away from rivers during their night-time foraging bouts. Previous research has reported sightings of *H. amphibius* ranging up to 0.3–30 km from a nearest known aquatic refuge^7,42,43,48^. In our study, the majority of *H. amphibius* movements occurred within 0.5–2 km and even the absolute maximum distance travelled away from the river that we recorded (4.7 km), fell short of the maximum distances found in other studies. Among-site differences in the distances travelled away from aquatic refugia by *H. amphibius* is likely related to the productivity and distribution of terrestrial resources^42^, distance to nearest watershed^48^, and the availability of temporary wallows in foraging areas^7^. This suggests that, much like other species, *H. amphibius* space use is context-dependent and the attributes of *H. amphibius* spatial ecology that we measured in Ruaha National Park should be replicated in local contexts where any spatial design strategies are being developed to better manage local *H. amphibius* populations.

Our resource selection function models and revisitation rate analyses identified that key *H. amphibius* foraging areas were highly structured around the river and use of these areas declined as the distance from the river increased. These results corroborate observations made elsewhere that *H. amphibius* are generally central place foragers^49^ and that the distance from rivers or water bodies significantly influences foraging decisions^12^. The *H. amphibius* tracked in our study invested more time in patches further from the river to compensate for increased travel costs. During the wet season, *H. amphibius* did not conform to central place foraging predictions because they are less likely to be energetically constrained by the higher availability and quality of the herbaceous layer^50,51^.

Past observations have suggested that *H. amphibius* habitat use is largely constrained to open habitats^42,43^. However, we found that *H. amphibius* also selected for woody habitats. The combination of *H. amphibius* being a large generalist herbivore and that their key foraging areas are in close proximity to the river, which reduces energetic travel costs, likely results in *H. amphibius* meeting their energetic demands without having to increase search or travel time to select specific habitats. This could potentially explain why *H. amphibius* used many of the available habitats less than expected. Floodplains are a key resource for *H. amphibius* because they are able to maintain grazing lawns with short-cropped, green foliose, an important resource for *H. amphibius*^3,26,42^, even at the peak of the dry season (Fig. 1b). *H. amphibius* only shift their habitat use when the grazing lawns in the floodplains were flooded.

The small home range size and obligate dependency of *H. amphibius* on aquatic refugia and floodplain food resources has important implications for mitigating crop raiding and human-*H. amphibius* conflict. Although we did not observe crop-raiding by *H. amphibius*, this behaviour has been reported along the periphery of the park^52^ and is a significant issue across the geographic range of *H. amphibius*^53,54^. The probability of crop raiding increases when farms are in close proximity to water sources or near *H. amphibius* access points to water sources^52^. Thus, by taking advantage of the limited movement of *H. amphibius* away from water sources, buffer zones around rivers or lacustrine environments could provide a low-cost solution to mitigate crop raiding and retaliatory killing of *H. amphibius*. Such riparian buffers are also well known in other management contexts to provide the added value of protecting watershed resources and providing wildlife corridors^55,56^. Alternatives, such as fencing farms with electric fences^54^ are costly and frequently not a viable solution for rural areas. Furthermore, the resident behaviour of *H. amphibius* should make the identification of consistent access points viable. However, this may be more complicated for the subset of the population that displays migratory behaviour and visit multiple aquatic refugia throughout the year. The implementation of buffer zones around rivers may also afford protection to important floodplain habitats from livestock, thereby reducing potential competition between livestock and *H. amphibius*. Increased grazing pressure by livestock in productive floodplain habitats can increase potential competition with *H. amphibius* by reducing food availability^2,57^. Overall, the patterns of *H. amphibius* space use that we observed underscore the value of riparian buffer zones as a tool to both minimize human-*H. amphibius* conflict^53^ and protect at-risk *H. amphibius* populations.

The patterns we observed in the Ruaha watershed reveal clear variation in the movement of different *H. amphibius* life history stages and provides the first evidence of large-scale male dispersal in this species. Although circumstantial, the only direct evidence of dispersal in *H. amphibius* is from a single female in South Africa^7^. The larger home range size of large sub-adult male *H. amphibius* can be attributed to the elevated levels of migratory and exploratory activities of this life stage – patterns that were clearly evident in the movement mode characterizations assigned to these individuals. Other observation-based studies of *H. amphibius* have previously noted that it is common for large sub-adults to be excluded from pools through aggressive interactions^11,58^. By contrast to the elevated mobility of the large sub-adult males, small sub-adult males held much smaller home ranges that were similar to the home range size of dominant male *H. amphibius*, affirming that these smaller sub-adult males are tolerated in the social groups controlled by dominant males.

We observed a very clear structuring influence of flow regime on certain elements of *H. amphibius* movement. A number of observational studies have noted that *H. amphibius* dispersal coincides with peak river flow^11,59^. However, in addition to down-stream migration during the wet season, we also observed migrations of large sub-adult males during the driest parts of the year when river flow was low (which included some of the wetting season). During this time, these large sub-adult males moved upstream away from downstream areas of the river that were drying most severely (Fig. 6a,b). During the dry season, the Great Ruaha River dries by approximately 60%^19^, thereby greatly reducing available water sources, which results in large aggregations forming in the remaining river pools (e.g. up to 95 individuals in a single river pool)^5,11^. Under these conditions, aggression and competition are exaggerated, which may be forcing these large sub-adult males to disperse under less than ideal environmental conditions. This would seem to explain why the large-scale movements that we observed were diffuse and uncoordinated, which are characteristics of avoidance-driven migration^60,61^. Furthermore, we posit that the spatial scale of these movement patterns may be greatly exacerbated by altered river hydrology because of the effects of reduced water availability on conspecific competition. By contrast, in less water stressed environments where the availability of suitable river pools is not limited, we suggest that migration behaviour would occur at smaller spatial scales and there would be less movement between multiple unsuitable pools. These hypotheses require exploration through future tracking studies in less water-stressed environments.

If food availability was a more dominant mechanism driving the observed migratory behaviours of large sub-adult males, we alternatively might have expected to find distinct seasonal movements more in accordance with the forage maturation hypothesis^62,63^. No such connections were observed in this study system. Furthermore, *H. amphibius* did not increase the distance they travelled away from the river when resources were limited during the dry season (as predicted by central place foraging theory). This suggests that food resources are not driving increased ranging behaviour, which supports our hypothesis that water availability, and not food resources, is driving the observed large-scale movements along the river.

Many climate forecasts for parts of sub-Saharan Africa predict prolonged periods of drought and diminished river flow^64,65^. These effects may be amplified by increases in anthropogenic water abstraction, such as those associated with expansion of agriculture. Based on the patterns in our tracking results observed across more and less dry periods, we suggest that river drying would most directly affect large sub-adult male *H. amphibius*, forced by the drying to undertake avoidance-driven migration to escape competition and aggression. For the other males and females, other density-dependent effects, induced by drying, may be more important. These density-dependent effects include: (1) elevated disease transmission rates resulting from increased aggregation sizes^66^, (2) increased mortality from aggressive interactions^11^, and (3) increased feeding competition around water sources with large aggregations. The energetically and physiologically stressful large-scale movements of large sub-adult male *H. amphibius* and these density-dependent effects together present significant challenges that in conjunction with even moderate human disturbances can lead to significant *H. amphibius* population declines^67^.

While connections between human-driven landscape aridification and *H. amphibius* behaviour and population health are critically important and timely to better understand, we caution against over-interpretation of the patterns we observed between seasonal drying in the Great Ruaha River and *H. amphibius* spatial ecology. Properly understanding these connections will require more tracking research on *H. amphibius* populations in less water limited regions as a point of comparison to these results as well as further longitudinal tracking work of *H. amphibius* in contexts that may be undergoing long-term drying.

Previous studies have shown that *H. amphibius* vector terrestrially-derived nutrients across ecosystem boundaries, thereby significantly shaping regional ecosystem ecology and biodiversity^4,14,68,69^. The observations we contribute here provide a clear opportunity to understand, in a more spatially-explicit and quantitative fashion, the role that *H. amphibius* plays in the structure and functioning of the terrestrial and aquatic habitats with which they interact. Stears *et al*.^5^ found that in the Great Ruaha River, river pools that maintain low-densities of *H. amphibius*, or no *H. amphibius* at all, act as important source pools during the dry season because of their ability to maintain aquatic biodiversity (no eutrophication due to low dung/nutrient inputs from *H. amphibius*). Frequently, *H. amphibius* do not inhabit smaller river pools because these pools do not provide *H. amphibius* with suitable protection from the sun or predators^11^. However, during the dry season when competition for suitable river pools is high, we observed large sub-adult male *H. amphibius* frequently being forced to inhabit relatively small, unsuitable river pools, that were previously unoccupied by *H. amphibius*. A single *H. amphibius* can egest ~5 kg of organic matter per day^4^, thus even relatively short residency by a single *H. amphibius* within these smaller river pools can result in eutrophic conditions and aquatic biodiversity loss^5^. Therefore, the frequent movement of large sub-adult males between multiple river pools, as a result of exaggerated levels of competition and aggression caused by the river drying, has the potential to greatly reduce the number of available source pools that may be important in shaping local patterns of aquatic species abundance and diversity within the Great Ruaha River.

A critically important caveat of this research is that we were not able to track the movements of female *H. amphibius*. Females were a major contributor to the increase in *H. amphibius* densities that we observed in riverine pools in the dry season. Dominant males allow females to freely enter their pools because it increases potential mating opportunities. Thus, we predict that when females move to a new pool, it is likely that they will set up residency in these pools, unlike the large sub-adult males that directly compete with dominant males. Future investigation that include tracking of females will be required to complete a portrait of the spatial ecology of this species, to better understand how the female population is influenced by hydrological variation, and to connect these observations to their effects on ecosystem ecology as well as to better inform *H. amphibius* management. In our study, we also only tracked individuals between 18h00 and 06h00, which precludes any opportunity to record any potential daytime activity or may have caused us to miss individuals that either leave or return to their pools before or after the start and ending periods for location fixes. Our camera trap and *in situ* observations suggested that daytime activities, however, were extremely limited. Manual inspection of GPS locations from tracked individuals also suggested that all movement paths started and ended in close proximity to the river. Based on these observations, we suggest that our sampling procedure captured the majority of *H. amphibius* movements in this population.

## Conclusions

Our results provide a first view of how male *H. amphibius* use their environment. They highlight the clear influence that life stage and hydrological regime have on the movement ecology of *H. amphibius* males. In particular, during the dry season, we observed significant (~15 km) upstream movement by large sub-adult males. These movements, coupled with other secondary stressors resulting from crowding of females and other life stages of males may have deleterious effects on *H. amphibius* populations in increasingly water-stressed contexts. Collectively, the movements we describe and quantify here provide important insight into the spatial scale and the potential degree of connectivity provided by this ecosystem-linking species. Furthermore, these results provide important insight into how *H. amphibius* populations can be most effectively conserved by proper water management policies (e.g. ensuring minimum environmental flow requirements), protecting riverine and lacustrine floodplains, anticipating upstream movements and ensuring connectivity between habitats in more flow sensitive rivers, and extending management zones to buffer rivers to reduce potential for *H. amphibius*-human conflict. While there is more to be learnt from future work that includes the tracking of females as well as *H. amphibius* populations in other hydrological contexts, these results collectively contribute critical spatially-explicit insights into the drivers that shape *H. amphibius* spatial ecology, how these behaviours shape their environment, and how we can better design strategies to improve the management of *H. amphibius* populations now and into the future.

## Supplementary information

Supplementary information

## Data Availability

The datasets generated during and/or analysed during the study are available from the corresponding author on reasonable request.
