# Supplementary material for: Spatial ecology of male hippopotamus in a changing watershed
Source: Sci Rep. 2019 Oct 28;9:15392. doi: 10.1038/s41598-019-51845-y (PMC6817855; doi:10.1038/s41598-019-51845-y)
Supplement: Supplementary file 1 — Supplementary information [file 41598_2019_51845_MOESM1_ESM.pdf]

Supplementary information for:

The spatial ecology of male hippopotamus in a changing watershed

Keenan Stears[1], Tristan A. Nuñez[2], Epaphras A. Muse[3], Benezeth M. Mutayoba[4], and Douglas J. McCauley[1]

Correspondence and requests for materials should be addressed to K.S.
email: keenanstears@ucsb.edu

Supplementary Methods S1

<u>Stationary GPS collar test.</u>

We conducted stationary tests of GPS collars to determine the fix-rate success (FRS) and location error (LE) associated with each collar over a four-day period. The individual collars were elevated by ~10 cm to mimic the height that the collars would be when attached to *Hippopotamus amphibius*. Each GPS collar was separated by 1 m and simultaneously placed in an open habitat without topographical or vegetation obstructions to ensure favourable satellite views. We calculated the FSR by dividing the number of collected fixes by the maximum number of fixes expected during the four-day period. LE was calculated for each positional fix by calculating the Euclidean distance between each of the collected GPS location fixes and the corresponding “true” collar location using the following formula:

$$LE = [\Delta x^2 + \Delta y^2]^{0.5} \qquad (1)$$

Where Δx and Δy are the differences between the collected and the ‘true’ x- and y co-ordinates respectively[1].

Supplementary Methods S2

Net squared displacement metrics to measure *Hippopotamus amphibius* displacement relative to their movements parallel to the river and perpendicular to the river.

We observed a large increase in the home range estimates for large-sub *H. amphibius*. These increases could be the result of *H. amphibius* moving greater distances away from the river (perpendicular), or alternatively, their movements could be focused parallel to the river. Thus, to quantify whether *H. amphibius* movements were parallel, or perpendicular from the river, we calculated the net squared displacement (NSD) of each *H. amphibius* (n = 10) location from its initial pool. We defined the initial pool as the location in which an individual *H. amphibius* was collared. For each location, we calculated two NSD values. The first value represents movement away from the river and the second reflects movement along the river. To calculate these two values, we used the snapPointsToLines function in the maptools package in R[2]. From this, we calculated the distance travelled perpendicular from the river as the snapped distance (i.e. distance of a location to the closet point on the river). The second NSD value, distance travelled parallel to the river, was measured as the distance from the snapped point on the river to its initial pool (see Fig. S4).

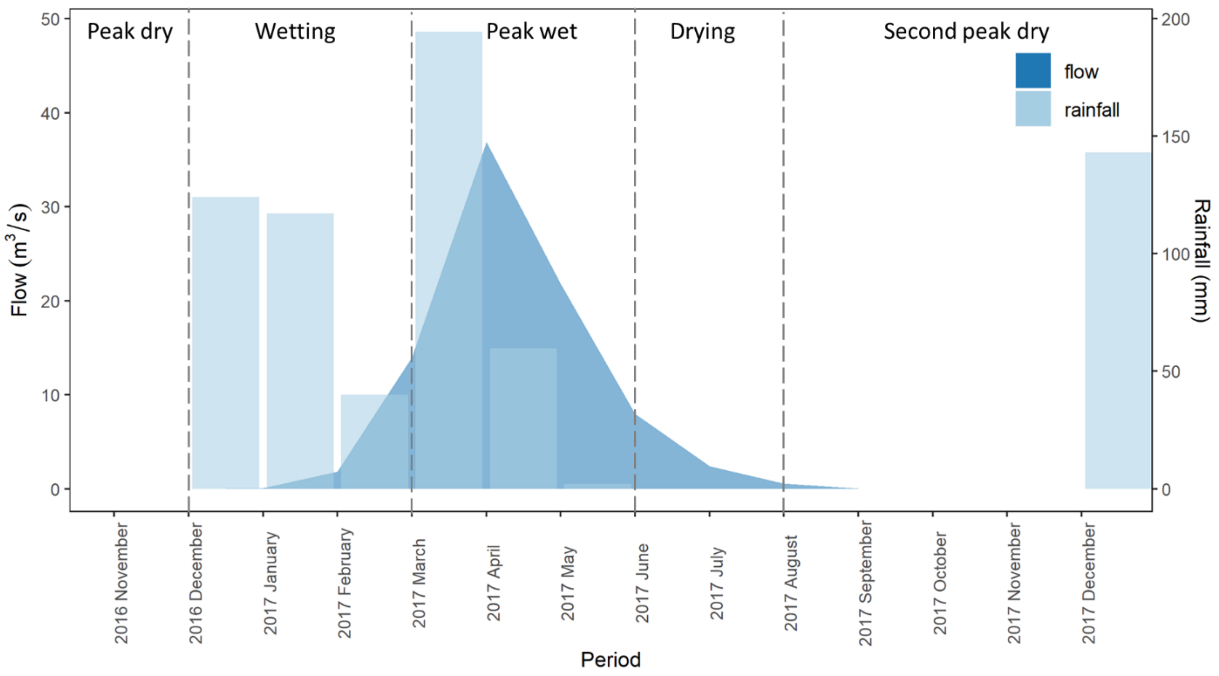

Figure S1: Monthly rainfall (mm) and the corresponding flow (m^3/s) for the Great Ruaha River, Ruaha National Park, Tanzania. Rainfall and river flow influence resource availability (terrestrial and aquatic) and were used to categorize the following seasonal periods: 1) Peak dry period: no rainfall and zero flow, 2) Wetting period: start of the rainy season with an increase in river flow only being observed towards the end of the wetting period, 3) Peak wet: peak rainfall and river flow, 4) Drying period: no rainfall and rapidly drying river, and 5) Second peak dry: no rainfall and zero flow.

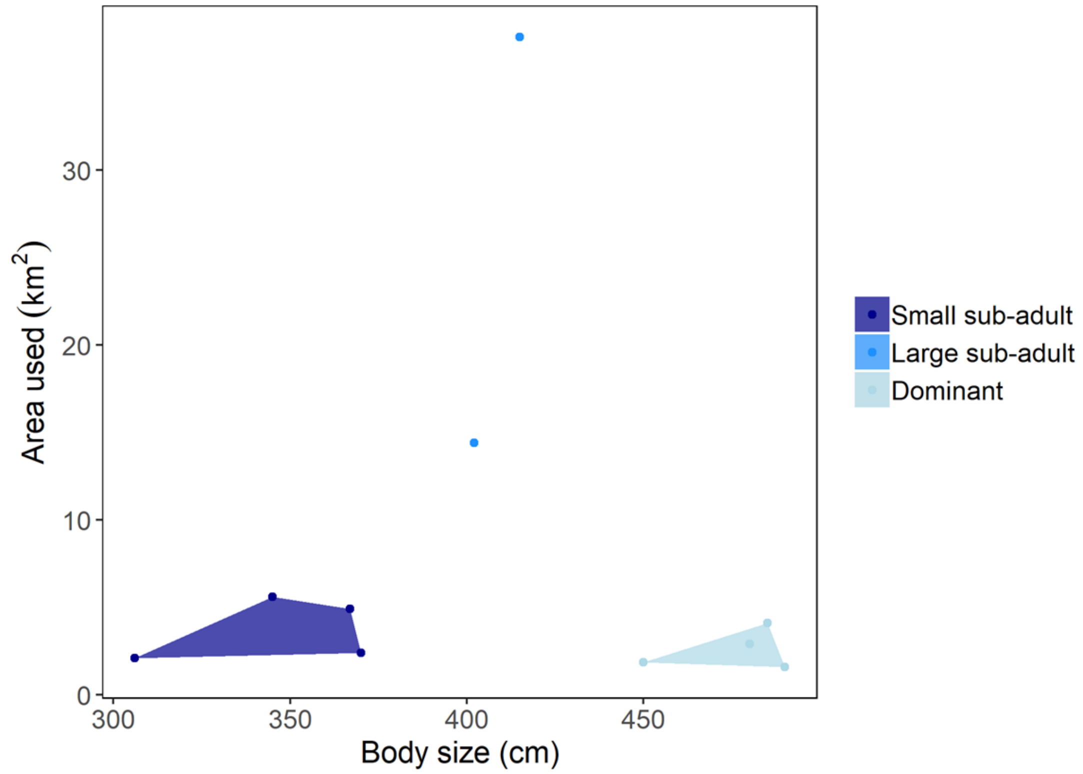

Figure S2: We used natural breaks in the relationship between individual *Hippopotamus amphibius* home range size and body length to categorize male *H. amphibius* into the following three life stage classes: small sub-adult male, large sub-adult male, and dominant male.

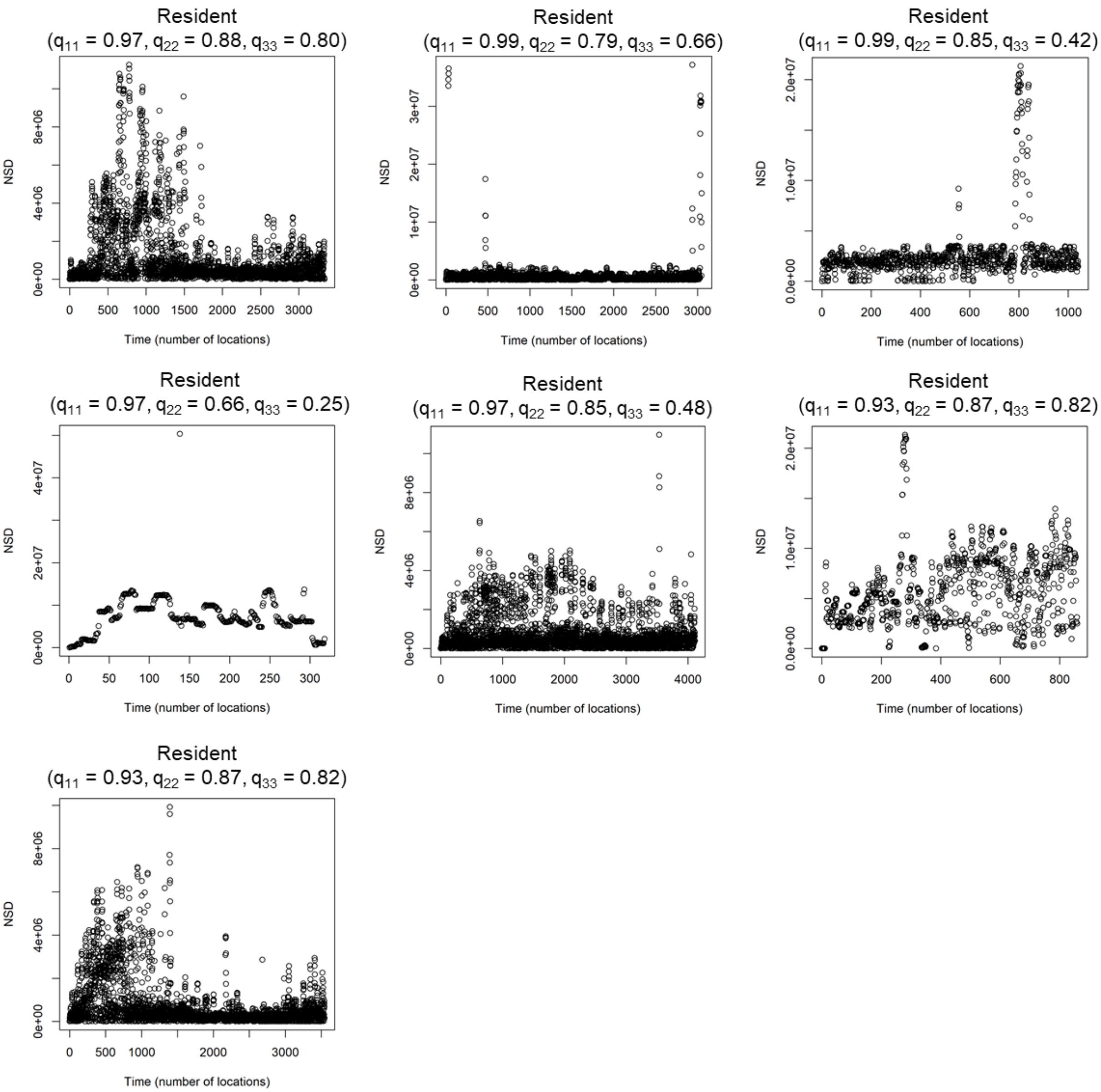

Figure S3: Net squared displacement values derived from the distance between the starting location and subsequent locations in a *Hippopotamus amphibius* movement path. These net squared displacement values in conjunction with switching probabilities (see main text) were used to characterize the different movement modes exhibited by *H. amphibius*. Switching probabilities for resident movement strategy when: $q_{22} \leq 0.90$ and $q_{33} \leq 0.90$.

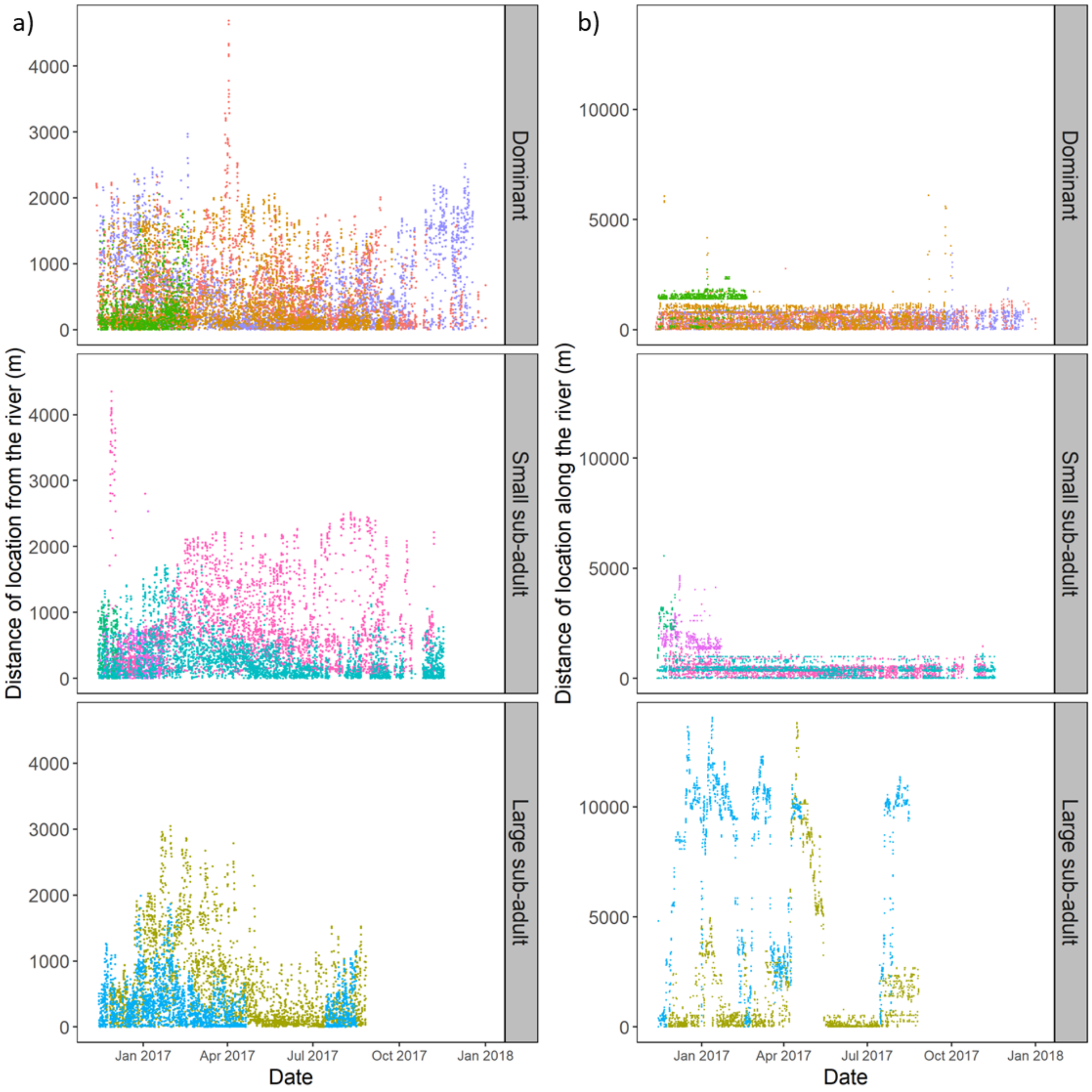

Figure S4: To assess *Hippopotamus amphibius* movement patterns in relation to the Great Ruaha River, we referenced *H. amphibius* locations with respect to: a) their distance away from the river (perpendicular to the river), and b) their distance along the river (parallel to the river) for each of the life stage categories. Colours denote individual *H. amphibius* and note differences in scale between panels a and b.

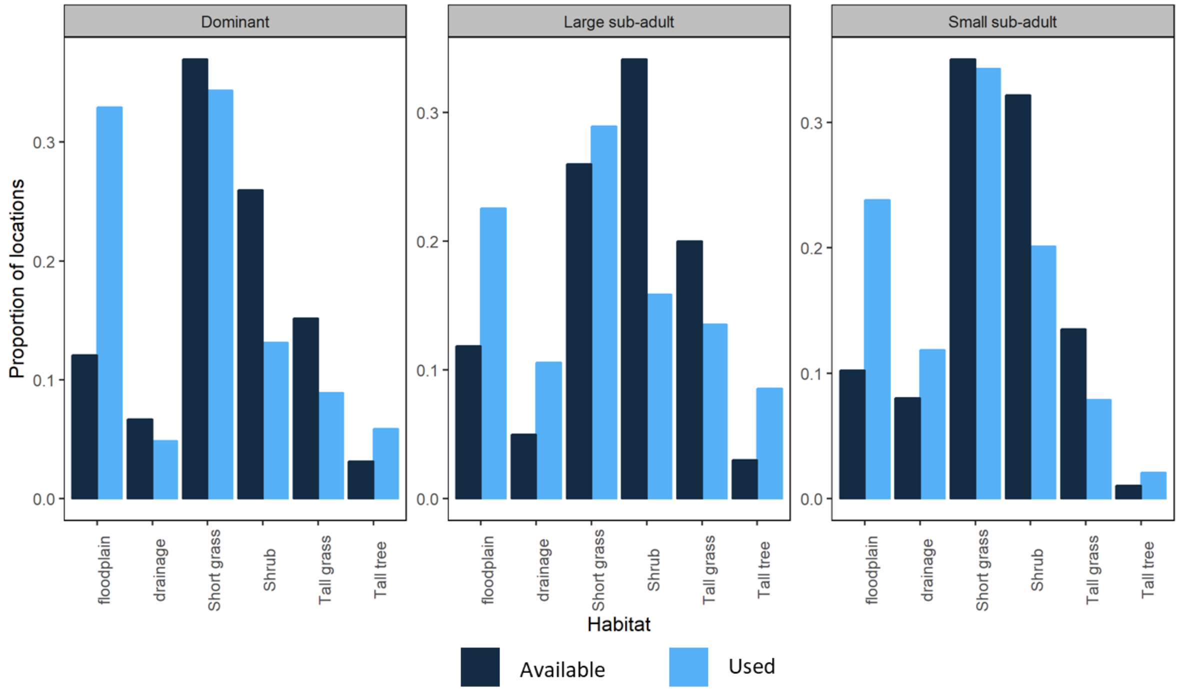

Figure S5: The proportion of habitat in the set of available locations and the proportion of habitat in the set of used locations (e.g., selection ratio for each habitat) for each of the *Hippopotamus amphibius* life stage categories. The habitat floodplain consistently had the highest positive selection ratios (preferred habitat) and was therefore used as the reference category in the resource selection function models (see main text). Floodplain always had positive selection ratios irrespective of how the data were categorized (e.g., combined by season, status, individual *H. amphibius* plots, or all location data combined).

Table S1: The duration that each collar remained on a *Hippopotamus amphibius* as well as the total number of fixes obtained. Collars that did not last throughout the study period fell off due to collars being submerged in water for extended periods of time.

	Sampling period														
	Peak dry	Wetting			Peak wet			Drying		Peak dry					
Collar ID	Nov	Dec	Jan	Feb	Mar	Apr	May	Jun	Jul	Aug	Sep	Oct	Nov	Dec	Number of fixes
1															3370
2															3052
3															2993
4															1043
5															318
6															3290
7															2685
8															4115
9															864
10															3554

Table S2: Model selection for seasonal resource selection function (RSF) models for dominant and small sub-adult male *Hippopotamus amphibius*. Best fit models were based on Akaike Information Criterion for small sample sizes (AICc) and Akaike weights (AICcWt). K is the number of estimated parameters and LL is the log-likelihood of each model. The highlighted text indicates the best fit model.

Season	Fixed structure	Random structure	K	AICc	ΔAICc	AICcWt	LL
Peak dry	Distance travelled from the river + habitat + slope	Slope and intercept	35	11568.81	0.00	1.00	-5748.61
	Slope + habitat	Slope and intercept	31	11608.18	39.37	0.00	-5772.47
	Distance travelled from the river + habitat	Slope and intercept	31	11625.00	56.19	0.00	-5780.88
	Habitat	Slope and intercept	27	11658.78	89.97	0.00	-5801.92
	Distance travelled from the river + slope	Slope and intercept	9	11796.03	227.22	0.00	-5888.96
	Distance travelled from the river	Slope and intercept	5	11844.91	276.10	0.00	-5917.43
	Slope	Slope and intercept	5	12014.89	446.08	0.00	-6002.42
Wetting	Distance travelled from the river + habitat + slope	Slope and intercept	35	15835.86	0.00	1.00	-7882.23
	Distance travelled from the river + habitat	Slope and intercept	31	15859.19	23.33	0.00	-7898.05
	Slope + habitat	Slope and intercept	31	15943.53	107.67	0.00	-7940.22
	Habitat	Slope and intercept	27	15949.00	113.14	0.00	-7947.08
	Distance travelled from the river + slope	Slope and intercept	9	15987.45	151.59	0.00	-7984.68
	Distance travelled from the river	Slope and intercept	5	16010.93	175.07	0.00	-8000.45
	Slope	Slope and intercept	5	16206.27	370.40	0.00	-8098.12
Peak wet	Distance travelled from the river + habitat + slope	Slope and intercept	35	11023.88	0.00	0.91	-5475.89
	Distance travelled from the river + habitat	Slope and intercept	31	11028.46	4.59	0.09	-5482.41
	Slope + habitat	Slope and intercept	31	11138.35	114.47	0.00	-5537.35
	Habitat	Slope and intercept	27	11146.34	122.46	0.00	-5545.54
	Distance travelled from the river + slope	Slope and intercept	9	11335.46	311.58	0.00	-5658.66
	Distance travelled from the river	Slope and intercept	5	11344.5	320.64	0.00	-5667.23
	Slope	Slope and intercept	5	11661.2	637.34	0.00	-5825.58
Drying	Distance travelled from the river + habitat + slope	Slope and intercept	35	6798.63	0	1.00	-3362.98
	Distance travelled from the river + habitat	Slope and intercept	31	6829.47	30.84	0.00	-3382.70
	Slope + habitat	Slope and intercept	31	6845.53	46.9	0.00	-3390.76
	Habitat	Slope and intercept	27	6862.07	63.44	0.00	-3403.25
	Distance travelled from the river + slope	Slope and intercept	9	6866.56	67.93	0.00	-3424.19
	Distance travelled from the river	Slope and intercept	5	6906.69	108.06	0.00	-3448.31
	Slope	Slope and intercept	5	7221.35	422.72	0.00	-3605.65

Table S3: Model selection for seasonal resource selection function (RSF) models for large sub-adult male *Hippopotamus amphibius*. Best fit models were based on Akaike Information Criterion for small sample sizes (AICc) and Akaike weights (AICcWt). K is the number of estimated parameters and LL is the log-likelihood of each model. The highlighted text indicates the best fit model.

Season	Fixed structure	Random structure	K	AICc	∆AICc	AICcWt	LL
Peak dry	Distance travelled from the river + habitat	Slope and intercept	31	7995.10	0.00	0.97	-3965.82
	Distance travelled from the river + habitat + slope	Slope and intercept	35	8002.36	7.26	0.03	-3965.25
	Habitat	Slope and intercept	27	8023.85	28.75	0.00	-3984.37
	Slope + habitat	Slope and intercept	31	8026.21	31.11	0.00	-3981.38
	Distance travelled from the river	Slope and intercept	5	8111.52	116.42	0.00	-4050.74
	Distance travelled from the river + slope	Slope and intercept	9	8118.01	122.90	0.00	-4049.94
	Slope	Slope and intercept	5	8183.71	188.61	0.00	-4086.83
Wetting	Distance travelled from the river + habitat + slope	Slope and intercept	35	21389.64	0.00	1.00	-10659.23
	Distance travelled from the river + habitat	Slope and intercept	31	21404.69	15.05	0.00	-10670.88
	Slope + habitat	Slope and intercept	31	21586.30	196.66	0.00	-10761.68
	Habitat	Slope and intercept	27	21630.19	240.55	0.00	-10787.74
	Distance travelled from the river + slope	Slope and intercept	9	21660.37	270.73	0.00	-10821.15
	Distance travelled from the river	Slope and intercept	5	21664.05	274.41	0.00	-10827.01
	Slope	Slope and intercept	5	22079.80	690.16	0.00	-11034.89
Peak wet	Distance travelled from the river + habitat	Slope and intercept	31	15500.20	0.00	0.92	-7718.55
	Distance travelled from the river + habitat + slope	Slope and intercept	35	15505.07	4.87	0.08	-7716.84
	Distance travelled from the river + slope	Slope and intercept	9	15533.32	33.12	0.00	-7757.61
	Distance travelled from the river	Slope and intercept	5	15536.48	36.28	0.00	-7763.22
	Slope + habitat	Slope and intercept	31	15712.65	212.45	0.00	-7824.77
	Habitat	Slope and intercept	27	15739.47	239.27	0.00	-7842.31
	Slope	Slope and intercept	5	15912.57	412.37	0.00	-7951.27
Drying	Distance travelled from the river + habitat + slope	Slope and intercept	35	8199.61	0	0.65	-4063.89
	Distance travelled from the river + habitat	Slope and intercept	31	8200.88	1.27	0.34	-4068.72
	Slope + habitat	Slope and intercept	31	8209.17	9.56	0.00	-4072.87
	Habitat	Slope and intercept	27	8235.78	36.17	0.00	-4090.35
	Distance travelled from the river	Slope and intercept	5	8316.41	116.8	0.00	-4153.18
	Distance travelled from the river + slope	Slope and intercept	9	8325.82	126.21	0.00	-4153.84
	Slope	Slope and intercept	5	8462.49	262.88	0.00	-4226.23

References:

1. Rempel, R. S., Rodgers, A. R. & Abraham, K. F. Performance of a GPS animal location system under boreal forest canopy. *J. Wildl. Manage*. 59, 543–551 (1995).
2. Bivand, R. & Lewin-Koh, N. maptools: tools for handling spatial objects. R package version 0.9-5. https://CRAN.R-project.org/package=maptools (2019).
